# Phytoplankton alpha diversity indices response the trophic state variation in hydrologically connected aquatic habitats in the Harbin Section of the Songhua River

**DOI:** 10.1038/s41598-020-78300-7

**Published:** 2020-12-07

**Authors:** Fanhua Meng, Zhenxiang Li, Lei Li, Feng Lu, Yan Liu, Xinxin Lu, Yawen Fan

**Affiliations:** 1 College of Life Science and Technology, Harbin Normal University, Harbin, 150025 China; 2 Key Laboratory of Biodiversity of Aquatic Organisms, Harbin Normal University, Harbin, 150025 China

**Keywords:** Ecology, Hydrology, Limnology

## Abstract

The relationship between biodiversity and ecological functioning is a central issue in freshwater ecology, but how this relationship is influenced by hydrological connectivity stress is still unknown. In this study we analyzed the dynamic of the phytoplankton alpha diversity indices and their relationships with trophic state in two hydrologically connected aquatic habitats (Jinhewan Wetland and Harbin Section of the Songhua River) in the Songhua River Basin in northeast China. We hypothesized that the phytoplankton alpha-diversity indices have the potential to provide a signal linking trophic state variation in hydrologically connected aquatic habitats. Our results showed the Cyanophyta and Bacillariophyta were abundant at most stations. T-test showed that phytoplankton alpha diversity indices varied significantly between rainy season and dry season. Trophic State Index recorded that a meso-trophic to eutrophic states of two connected habits during study period. Multivariate statistical analysis revealed that the dynamic of phytoplankton alpha diversity index was closely associated with trophic states change. Our result indicated that hydrological connectivity is a key factor influenced phytoplankton community assembly. In addition, it is beneficial to develop an integrated approach to appropriately describe and measure the trophic state variations of hydrologically connected aquatic habits in freshwater ecosystem.

## Introduction

Phytoplankton, like land plants that have chlorophyll to capture sunlight^1^, make up a vital component of primary production in aquatic ecosystems and play a significant proportion in food web dynamics, energy flow, and nutrient cycling^2–4^. Phytoplankton communities are responsive rapidly to environmental variation because of their short life cycle and sensitive to nutrient change^5,6^. As phytoplankton are vital in introducing energy to food webs, particular focus have been devoted to understanding the relationship between their diversity pattern and environmental variation^7^. Numerous researches of phytoplankton had been performed in rivers^2^, lakes^5,8^ and reservoirs^9^, where it is well known that local environmental conditions are essential components in explaining the phytoplankton diversity pattern^3,7^. The degree of physical and chemical variables in affecting phytoplankton alpha diversity indices is different in individual aquatic habitats. However, the relationships between alpha-diversity indices and environmental variables in hydrologically connected aquatic habitats are unclear. Phytoplankton alpha diversity indices and trophic states in one aquatic habitat can be influenced by other connected aquatic habitats through water flow, transporting both nutrients and organisms^2^. Thus, phytoplankton alpha diversity indices and ecological traits tend to be similar among interconnected aquatic habitats in freshwater and ocean systems^3,10,11^. In contrast, phytoplankton alpha diversity indices become less similar among aquatic habitats with little or no hydrological connectivity. Prior studies showed that in interconnected aquatic habitats, phytoplankton, particular motile species assemblages are strongly influenced by hydrological connectivity change^3,12^. For example, Katsiapi et al.^12^ noted that cyanobacterial alpha diversity was affected by directional hydrological connectivity and high dispersal rates, these result could be explained by individual motive ability. Meanwhile, the phytoplankton richness, evenness and abundance matrices were proved a relevant approach to reveal the ecological traits in recent river management events in hydrologically connected aquatic habits^13,14^. Understanding the underlying biotic indicator to explore community assemblage response to environmental change on interconnected aquatic ecosystem is a fundamental research objective in ecology, as well as vitally to help plan basin-wide monitoring and to implement effective management.

Anthropogenic eutrophication was the highest before the 2000s in Asia, when urban, agricultural and wastewaters entered rivers, lakes and wetlands directly, resulting in algal blooms consisted majorly of cyanobacteria and diatoms. After wastewaters were discharged, the concentration of nitrogen and phosphorus began to increase in many connected habits include urban wetlands and rivers. Rainfall has been proved to be intensified eutrophication in many hydrologically connected habits. In rainy season, the higher flow rate, resuspension, and intense winds resulting in changes in ecosystem function, integrity and phytoplankton diversity. For example, high water fellow velocity strongly suppress the promotion of the abundance of photoautotrophic algae, however, enhancing the relative abundance of diatoms. Low nitrogen condition mainly limited the reproduction of Chlorophyta species, however, Cyanophyta (*Anabaena* spp.) usually had a curvilinear response to decrease TN. As the local processes in hydrologically connected habits, including environmental filtering, biotic interactions and ecological drift, result in phytoplankton diversity distribution patterns, we hypothesized that phytoplankton alpha-diversity indices has the potential to provide a signal linking trophic state variation in hydrologically connected aquatic habitats.

The Songhua River Basin (41°42′–51°38′N, 119°52′–132°31′E) is located in NE China, where is a 20 km wide from east to west, and 1070 km long from north to south. The total area of the basin is about 5.6 × 10^4^ km^2^. Harbin City is one of the most developed areas in NE China. The 66-km long Harbin Section of the Songhua River, NE China's largest urban river, flows through the Harbin City. Harbin Section of the Songhua River is an important tourism and industrial area. Previous studies have shown that parts of this basin are severely contaminated by persistent organic pollutant^15^. The report on the state of the ecology and environment in China 2019 noted that the water quality of the Songhua River Harbin section has changed from oligotrophic to eutrophic. Eutrophication indicators of zooplankton were also found in the aquatic organisms in the Harbin Section of the Songhua River^16^. In the past decades, the wetlands in the Songhua River Basin have strong experienced fragmentation and shrinkage. Many farms were built concomitant with the loss of wetlands, meanwhile most urban wetlands are in eutrophication. In addition, continuous fertilizer used in agriculture activities and industrial wastewater discharge contributed to increasing trophic states and decreased water quality in the part of the basin. Plankton assemblages from some Songhua River areas were documented, indicating highly diversity and a wide distribution of potential environmental indicator species^16^. However, none of these studies showed that phytoplankton alpha diversity indices response to trophic state, and the phytoplankton community in hydrologically connected aquatic habits of the Harbin Section of the Songhua River has not been documented.

In this study, phytoplankton community; phytoplankton alpha diversity indices (Margalef, Shannon–weaver, Pielou, and Simpson); and Trophic State Index (TSI), were measured in interconnected aquatic habits: the Jinhewan wetland and the Harbin Section of the Songhua River. Correlation analysis (CA) and Spatial interpolation analysis (Inverse distance weighting, IDW) were applied to understand the response of phytoplankton alpha diversity indices to trophic state variation. We compared phytoplankton alpha diversity indices during rainy season and dry season (high hydrological connectivity and low hydrological connectivity, respectively). The purposes of the study are as follows: (1) to explored the Impacts of hydrological connectivity on phytoplankton communities and (2) to understand phytoplankton alpha diversity indices in relation to river trophic state. It would be beneficial to develop an integrated approach to appropriately describe and measure the trophic state variations of hydrologically connected aquatic habits in the Songhua River Basin.

## Methods

### Study area, sample collection and laboratory analyses

**Figure 1:**
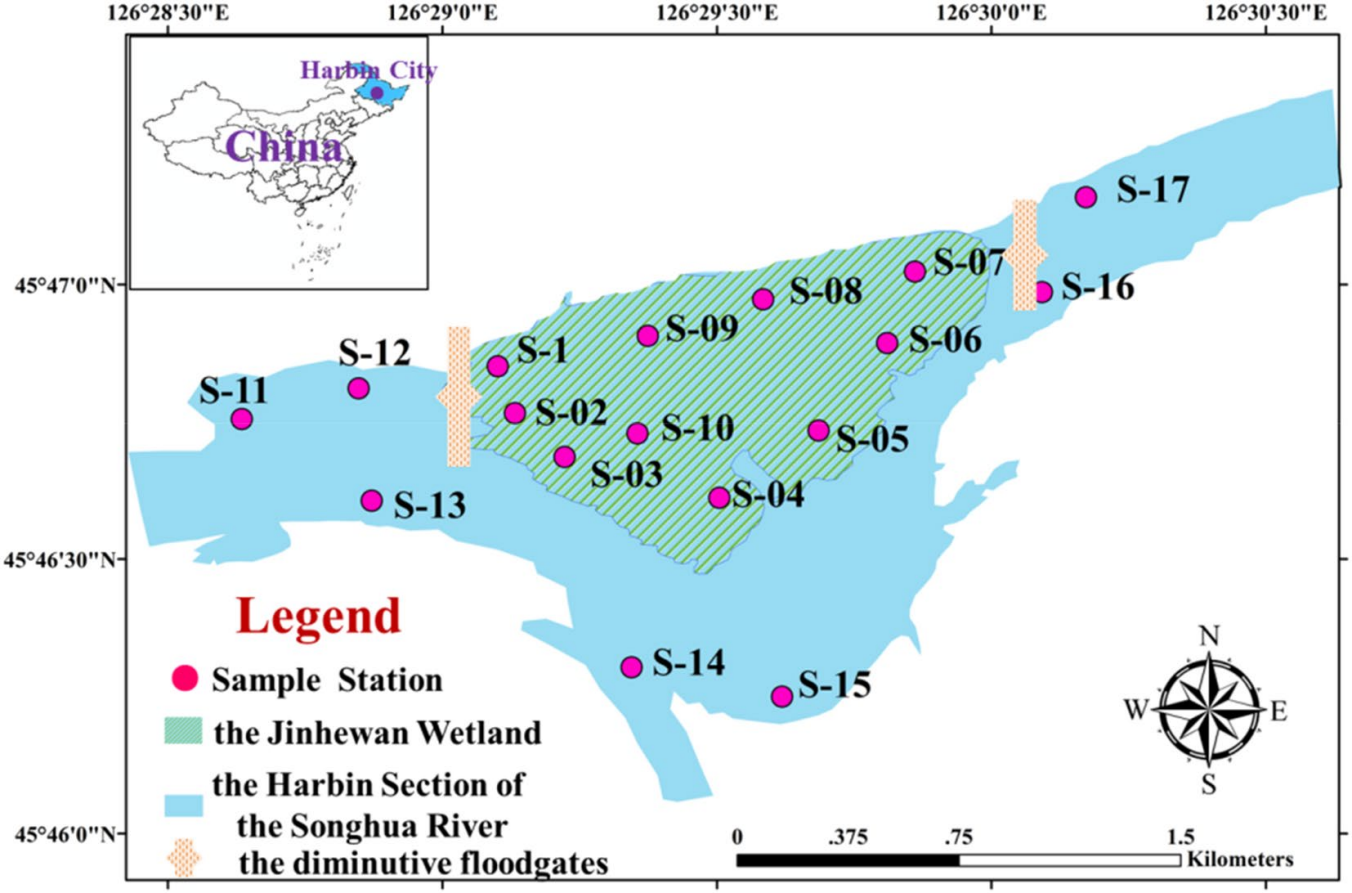
The location of sampling sties in study area. S1–S10 are located in Jinhewan Wetland, S11–S17 are located in the Harbin Section of the Songhua River. The map was generated by ArcGIS Desktop v10.2 (www.arcgis.com).

The Harbin Section of the Songhua River (HSSR) runs from Sanjia Village to Dadingzi Mountain^16^. Hydrological connectivity is controlled by diminutive floodgates between the JHWW and HSSR in study area. In addition, the connectivity of HSSR and JHWW is regulated by rainfall else, which higher rainfall lead to higher hydrological connectivity. During the connected period, the inflow coming from the HSSR flows through the JHWW and finally into the HSSR. The Jinhewan Wetland (JHWW) is located in the middle streams of the Harbin Section of the Songhua River, which is a demonstration area for the protection and restoration of aquatic ecosystems in Harbin, and is a vital part of the ecological project of the “Hectares of Songjiang Wetland and Long-stretching Ecological Corridor”^17^. The JHWW is the main inflow channel with a volume of 3 × 10^5^ m^3^ water, contributing more than 30% of the total inflows of HSSR. A phytoplankton study was carried out in rainy season (July, higher hydrological connectivity), and dry season (October, lower hydrological connectivity) in 2017 and 2018. A total of four samplings were performed during study period (July 2017, October 2017, July, 2018 and October 2018). In this study, we collected water samples from seventeen stations in two connected habits (Fig. 1); ten belong to the JHWW, seven to the HSSR. At each sampling station, geographic coordinates were determined using a Garmin Etrex GPS. The water samples were collected by 1 L water sampler. A total of 5 L of water samples were collected by plastic bottle preserved with 1% Lugol’s solution and refrigerated under dark conditions until laboratory analysis. Together with phytoplankton sampling, the 5 L samples for chemical analyses were collected. All of samples for chemical analyses were preserved in brown glass bottle at 4 °C portable refrigerator, immediate. A YSI multi-metric probe was used to measure physical factors in the field, such as water temperature (WT), dissolved oxygen (DO), conductivity (EC), Oxidation–reduction potential (ORP) and pH. Chemical factors such as total nitrogen (TN), total phosphorus (TP), chlorophyll a (Chl-a) and chemical oxygen demand (COD) were determined by Chinese national standards for water quality in laboratory within 24 h^18^. All of environmental parameters were measured with 3 replicates. The samples for phytoplankton analysis were sedimented for 24 h and concentrated to 50 mL^18^. Phytoplankton was qualitative and quantitatively analysis in a counting chamber with a compound microscope (Imager A2, Zeiss, Germany) at × 400 magnification. Phytoplankton species were identified based on morphology^19^.

### Alpha diversity indices

Phytoplankton alpha diversity indices were evaluated using the Shannon–Weaver diversity index, the Margalef index, Pielou evenness index, and Simpson index.

Shannon–Weaver index:

$$H^{\prime} = - \mathop \sum \limits_{i = 1}^{s} Pi \times \ln Pi$$

Margalef index:

$$H = \left( {S - 1} \right)/\ln N$$

Pielou evenness index:

$$J = H^{\prime } /\ln S$$

Simpson index:

$$D = 1 - \mathop \sum \limits_{i = 1}^{s} Pi^{2}$$

where N is the total number of all species in the sample; S is the total species in the sample; ni is the total individual numbers in species i.

### Multivariate statistical analysis

In present study, 10 physicochemical factors were considered for multivariate statistical analysis, including WT, DO, EC, pH, ORP, Tur., Chl-a, TN, TP and COD. All 10 factors and diatom data were normalized by [log10(x + 1)] transformation. Comprehensive trophic state index (TSI) was used to describe the trophic status^20^. The equations for TSI are as follows:

$${\text{TSI}}\left( {{\text{TN}}} \right) = 10\left( {5.453 + 1.694\,\ln \,{\text{TN}}} \right)$$

$${\text{TSI}}\left( {{\text{TP}}} \right) = 10\left( {9.436 + 1.642\,\ln \,{\text{TP}}} \right)$$

$${\text{TSI}}\left( {\text{Chl-a}} \right) = 10\left( {2.5 + 1.086\,\ln \,{\text{Chl-a}}} \right)$$

$${\text{TSI}}\left( {{\text{COD}}} \right) = 10\left( {1.2 + 1.566\,\ln \,{\text{COD}}} \right)$$

$${\text{TSIM}}\left( \sum \right) = \left[ {{\text{TSI}}\left( {{\text{TN}}} \right) + {\text{TSI}}\left( {{\text{TP}}} \right) + {\text{TSI}}\left( {{\text{COD}}} \right) + {\text{TSI}}\left( {\text{Chl-a}} \right)} \right]/4$$

Evaluation standard: 0 < TSIM ≤ 30 oligotrophic, 30 < TSIM ≤ 50 mesotrophic, TSIM > 50 eutrophic, 50 < TSIM ≤ 60 light eutrophic, 60 < TSIM ≤ 70 middle eutrophic, TSIM > 70 high eutrophic.

**Figure 2:**
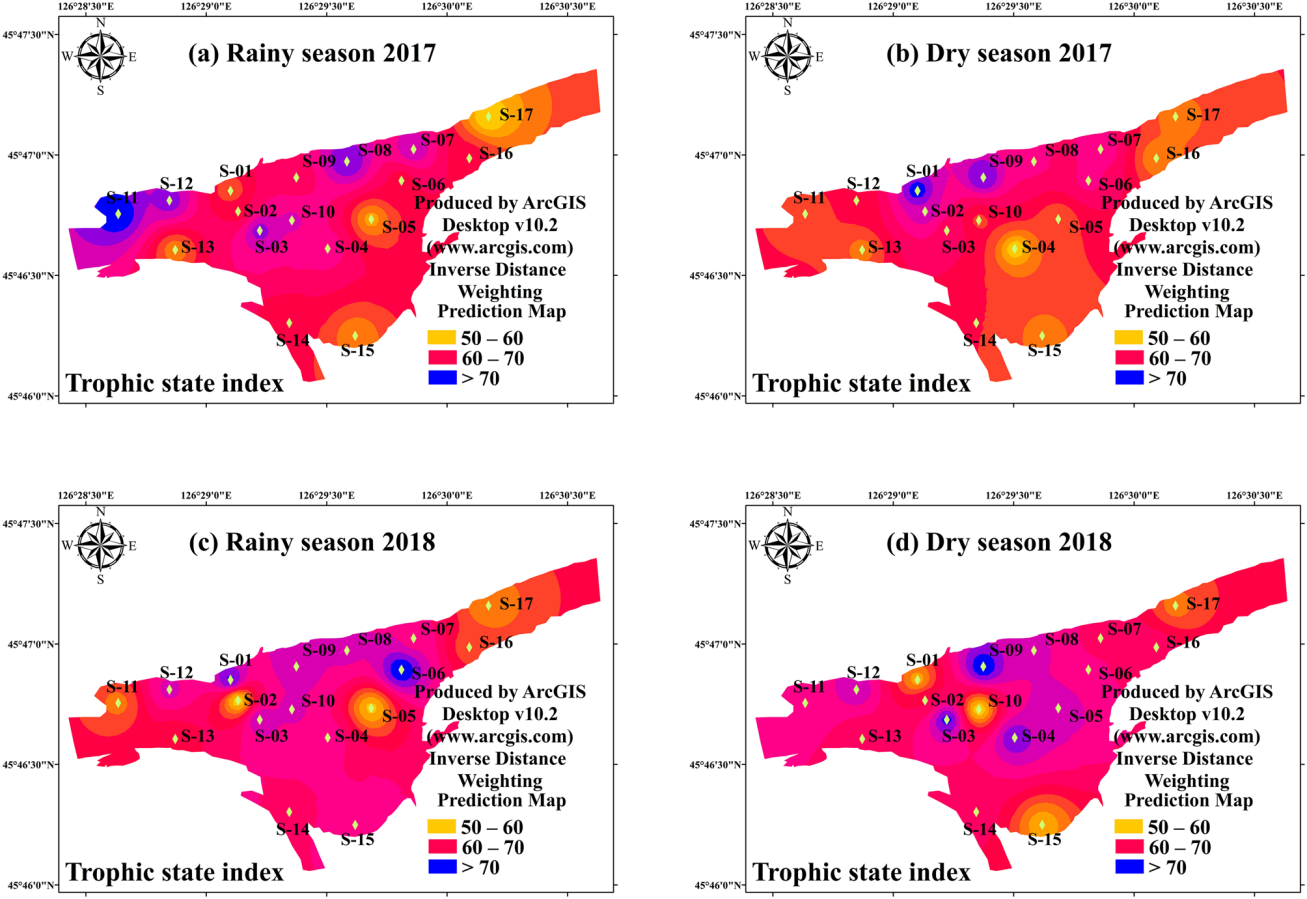
The spatial distribution of Trophic State Index in study period. (**a**) Rainy season (July 2017); (**b**) Dry season (October 2017); (**c**) Rainy season (July 2018); (**d**) Dry season (October 2018). The interpolation map was constructed by ArcGIS software using the Inverse Distance Weighting method. The maps were created using software ArcGIS Desktop v10.2 (www.arcgis.com).

**Figure 3:**
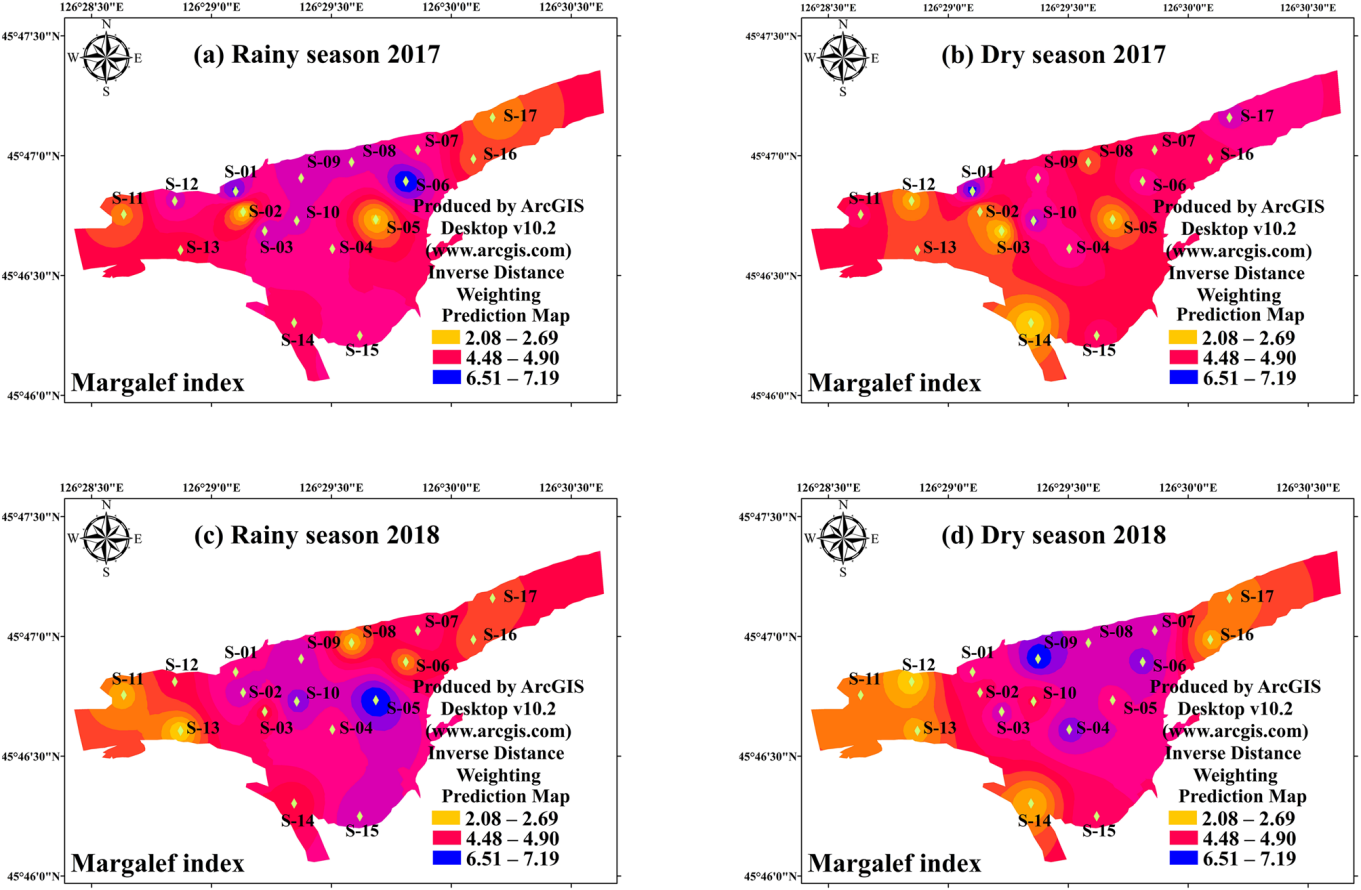
The spatial distribution of phytoplankton Margalef index in study period. (**a**) Rainy season (July 2017); (**b**) Dry season (October 2017); (**c**) Rainy season (July 2018); (**d**) Dry season (October 2018). The interpolation map was constructed by ArcGIS software using the Inverse Distance Weighting method. The maps were created using software ArcGIS Desktop v10.2 (www.arcgis.com).

**Figure 4:**
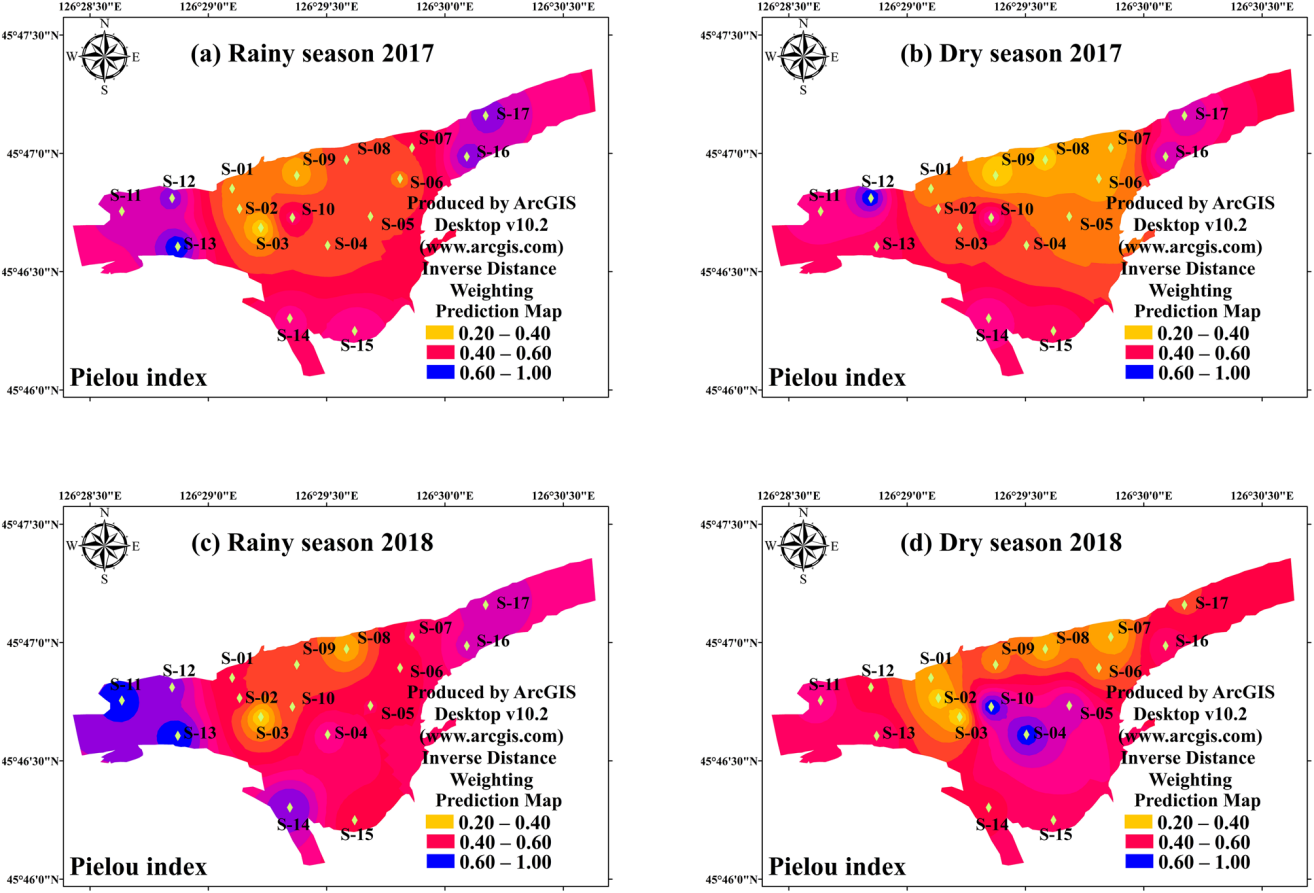
The spatial distribution of phytoplankton Pielou evenness index in study period. (**a**) Rainy season (July 2017); (**b**) Dry season (October 2017); (**c**) Rainy season (July 2018); (**d**) Dry season (October 2018). The interpolation map was constructed by ArcGIS software using the Inverse Distance Weighting method. The maps were created using software ArcGIS Desktop v10.2 (www.arcgis.com).

**Figure 5:**
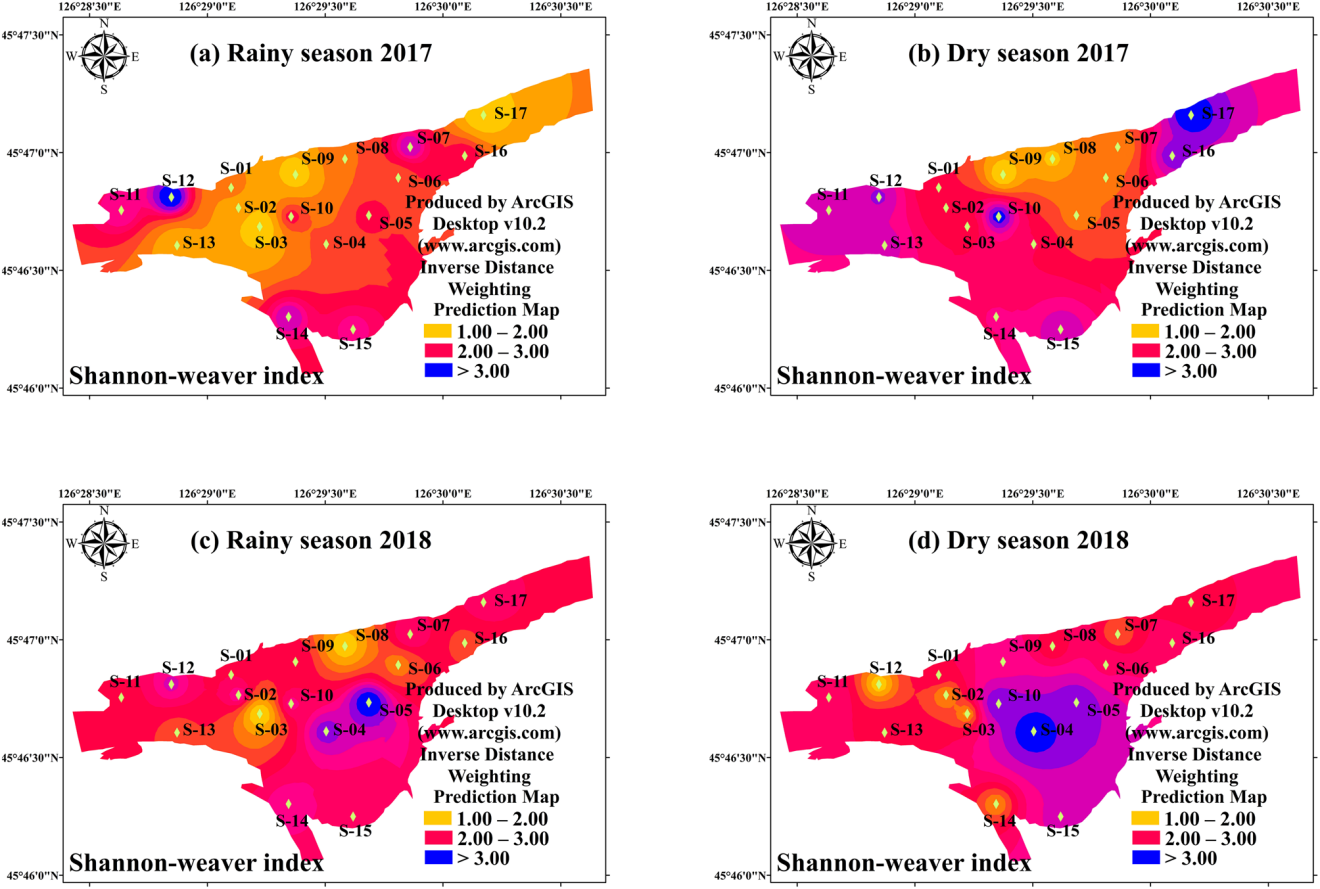
The spatial distribution of phytoplankton Shannon–Weaver index in study period. (**a**) Rainy season (July 2017); (**b**) Dry season (October 2017); (**c**) Rainy season (July 2018); (**d**) Dry season (October 2018). The interpolation map was constructed by ArcGIS software using the Inverse Distance Weighting method. The maps were created using software ArcGIS Desktop v10.2 (www.arcgis.com).

**Figure 6:**
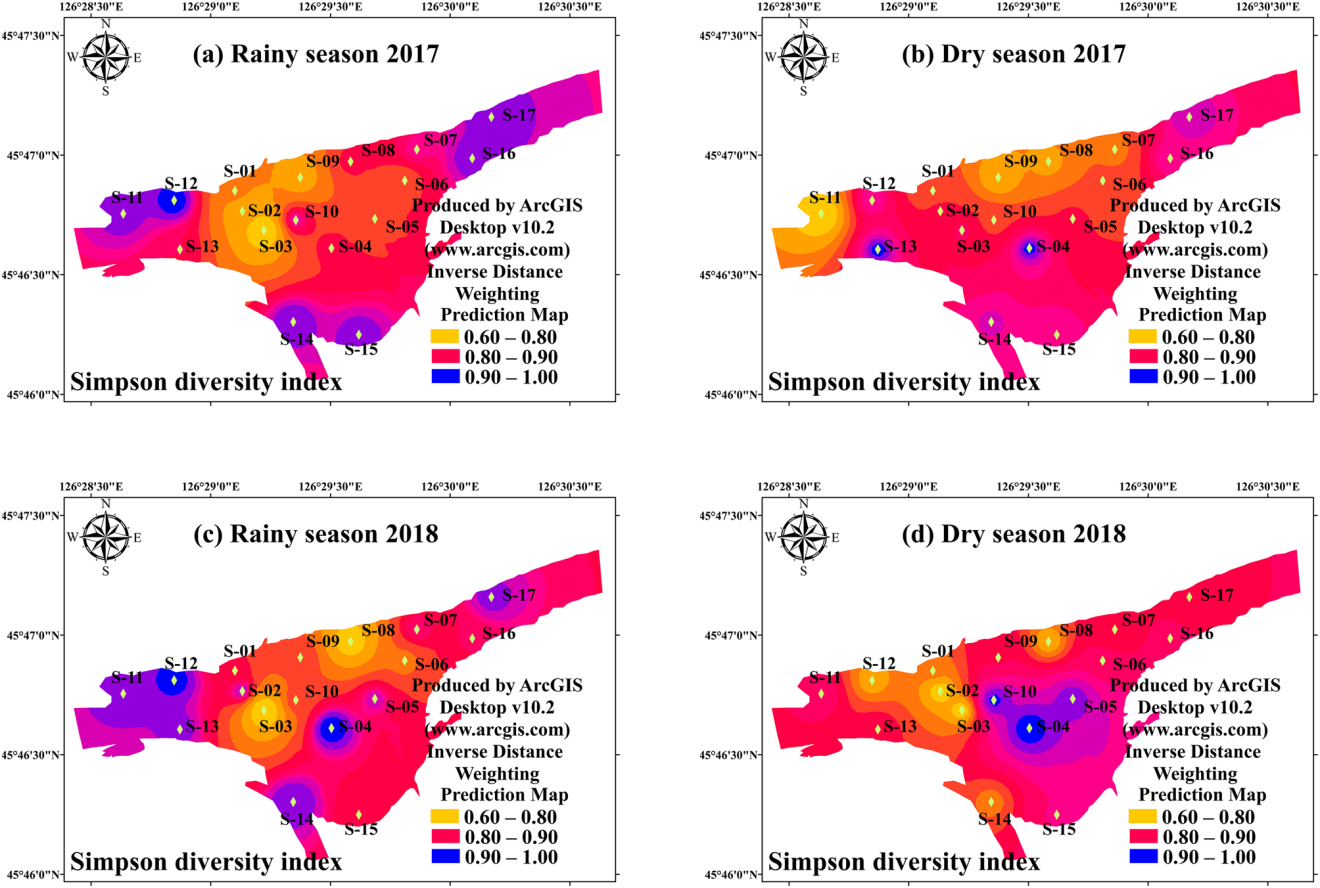
The spatial distribution of phytoplankton Simpson diversity index in study period. (**a**) Rainy season (July 2017); (**b**) Dry season (October 2017); (**c**) Rainy season (July 2018); (**d**) Dry season (October 2018). The interpolation map was constructed by ArcGIS software using the Inverse Distance Weighting method. The maps were created using software ArcGIS Desktop v10.2 (www.arcgis.com).

Inverse distance weighting (IDW) interpolation, which assumes that things that are close to one another are more alike than those which are farther apart, is widely performed to explain spatial variation and distribution of organic pollution and eutrophication in freshwater and ocean ecosystem^21,22^. The IDW method with weighting power of 2.0 was implemented to illuminate spatial variations of phytoplankton alpha diversity indices and TSI in this study. A good agreement between measured line and predicted line with the acceptable mean error (< 0.05) and Log (x + 1) transformed were conducted by ArcGIS, indicating that IDW interpolation was effectively applied in present study. IDW interpolation with weighting power of 2.0 was used to elucidate spatial variations of phytoplankton alpha diversity and TSI. Sampling map and IDW interpolation maps (Figs. 1, 2, 3, 4, 5, 6) were created using ArcGIS Desktop v10.2 (www.arcgis.com). Independent-samples T test (T-test) and correlation analysis (CA) was performed to identify the difference of environmental factors, which were conducted by SPSS 22.0.

## Results

### Environmental characteristics and trophic states

Six environmental variables, Water temperature, pH, Conductivity, Total nitrogen, Total phosphorus and Chemical oxygen demand showed significant differences (*p* < 0.05 or *p* < 0.01) across the two hydrological period, whereas four variables, Conductivity, pH, Chl-a and Chemical oxygen demand concentration, were significantly different (*p* < 0.05 or *p* < 0.01) in interconnectivity habits (Table 1). DO, EC, pH, ORP, Chl-a, TN, and COD were generally high during the dry season with concentrations increasing from the samples of river to wetland. These finding indicated that hydrological connectivity was a key factor in influencing the environmental variables variation in interconnected habits. An analysis of the spatial and temporal variations of total TSI (the combination of the TSI for TP, TN, COD, and Chl-a) was performed (Fig. 2). TSI indicated that trophic states of the study area ranged from mesotrophic to highly eutrophic (Table 1, Fig. 2). There was significant change between rainy season and dry season of the total TSI (Table 1) (*p* < 0.01). The mean TSI in rainy season was 56 ± 8, significantly lower than that in dry season (mean TSI 63 ± 7) (*p* < 0.01). As a whole, in dry season, the eutrophication state was more serious in rainy season, when a high trophic state occurred in widespread areas of the Harbin Section of the Songhua River. Based on environmental variables pattern and TSI, we found that hydrological connectivity was a vital component of ecological process that influenced the trophic state in interconnected habits of Songhua River Basin.

**Table 1:** The temporal and spatial variation of environmental parameters and TSI during study period.

	WT (°C)	DO (mg/L)	EC (μs/cm)	pH	ORP (mv)	Tur. (NTU)	Chl-a(μg/L)	TN (mg/L)	TP (mg/L)	COD (mg/L)	TSI
Rainy season	26.24 ± 1.30	1.10 ± 0.68	208.03 ± 13.58	9.44 ± 0.61	381.74 ± 22.28	36.76 ± 15.04	9.73 ± 6.63	0.40 ± 0.33	0.38 ± 0.15	14.16 ± 9.10	56 ± 8
Dry season	10.50 ± 1.86	2.43 ± 1.16	253.19 ± 66.03	9.52 ± 0.76	386.07 ± 23.05	33.62 ± 10.08	9.74 ± 9.56	1.55 ± 0.83	0.26 ± 0.16	19.73 ± 9.04	63 ± 7
Wetland	18.59 ± 8.06	1.81 ± 1.95	216.32 ± 26.48	9.77 ± 0.64	381.97 ± 25.89	35.05 ± 12.70	13.21 ± 9.08	1.11 ± 0.88	0.32 ± 0.16	21.06 ± 8.2	63 ± 7
River	18.05 ± 7.98	1.71 ± 1.11	251.04 ± 71.03	9.05 ± 0.52	386.67 ± 17.94	35.38 ± 14.17	4.77 ± 2.09	0.92 ± 0.80	0.33 ± 0.17	11.11 ± 7.81	56 ± 8
2017	17.99 ± 9.26	0.89 ± 0.49	260.19 ± 61.02	9.22 ± 0.75	387.04 ± 23.38	23.37 ± 3.77	9.55 ± 6.88	1.25 ± 0.97	0.37 ± 0.19	17 ± 10.22	61 ± 10
2018	18.75 ± 0.65	2.65 ± 0.95	201.04 ± 9.55	9.73 ± 0.52	380.76 ± 22.31	46.60 ± 7.61	9.92 ± 9.38	0.70 ± 0.61	0.27 ± 0.12	16 ± 8.67	57 ± 7
**T-test**											
Rainy × Dry	*p* < 0.01	*p* < 0.01	*p* < 0.01	*p* > 0.05	*p* > 0.05	*p* > 0.05	*p* > 0.05	*p* < 0.01	*p* < 0.05	*p* < 0.05	*p* < 0.01
Wetland × River	*p* > 0.05	*p* > 0.05	*p* < 0.05	*p* < 0.05	*p* > 0.05	*p* > 0.05	*p* < 0.01	*p* > 0.05	*p* > 0.05	*p* < 0.01	*p* < 0.01
2017 × 2018	*p* > 0.05	*p* > 0.05	*p* > 0.05	*p* > 0.05	*p* > 0.05	*p* < 0.05	*p* > 0.05	*p* < 0.05	*p* < 0.05	*p* > 0.05	*p* > 0.05

### Temporal-spatial variations of phytoplankton diversity indices

A total of 221 species of phytoplankton belonged to 8 phyla and 79 genera were identified, including Chlorophyta (43.89%), Bacillariophyta (26.24%), Cyanophyta (16.74%), Euglenophyta (9.05%), Pyrrophyta (2.26%), Cryptophyta (0.90%), and others (0.45%). Phytoplankton abundance ranged from 0.13 × 10^6^ ind./L to 244.67 × 10^6^ ind./L during the study period. There was no significant variation of phytoplankton abundance in different hydrological period (*p* > 0.05) (Table 2). Nevertheless, phytoplankton abundance showed significantly spatial heterogeneity in the two habitats (*p* < 0.01), with higher values in sample sites of wetland and lower values in river (Table 2). Overall, Cyanophyta and Bacillariophyta were co-dominant in study period, which contributed more than 80% total phytoplankton abundance (mean 26.37 × 10^6^ ind./L and 12.87 × 10^6^ ind./L, respectively) (Table 2).

**Table 2:** The temporal and spatial variation of phytoplankton alpha diversity indices and phytoplankton community structure during study period.

	Margalef	Peilou		Shannon–Weaver	Simpson	Abundance (× 10^6^ ind./L)	Bacillariophyta(× 10^6^ ind./L)	Cyanophyta (× 10^6^ ind./L)	Euglenophyta(× 10^6^ ind./L)	Chlorophyta(× 10^6^ ind./L)	others(× 10^6^ ind./L)
Rainy season	4.79 ± 1.33	0.71 ± 0.15		2.26 ± 0.32	0.82 ± 0.08	31.77 ± 31.59	6.2 ± 5.5	21.53 ± 27.71	0.69 ± 1.26	2.37 ± 2.45	0.21 ± 0.32
Dry season	4.76 ± 0.93	0.57 ± 0.18		1.8 ± 0.50	0.68 ± 0.15	55.94 ± 68.62	19.53 ± 25.34	31.22 ± 55.89	0.61 ± 1.64	2.84 ± 4.17	0.26 ± 0.55
Wetland	4.97 ± 1.13	0.73 ± 0.13		1.94 ± 0.51	0.73 ± 0.13	71.92 ± 51.36	19.77 ± 22.89	44.70 ± 50.31	0.95 ± 1.32	4.18 ± 3.71	0.41 ± 0.53
River	3.67 ± 0.87	0.78 ± 0.12		2.15 ± 0.39	0.79 ± 0.14	3.76 ± 3.83	3.00 ± 3.14	0.19 ± 0.28	0.21 ± 0.65	0.33 ± 0.35	0.03 ± 0.04
2017	4.44 ± 1.17	0.65 ± 0.20		2.04 ± 0.50	0.76 ± 0.15	55.82 ± 65.15	8.92 ± 10.93	41.97 ± 56.22	0.50 ± 0.89	2.04 ± 2.19	0.14 ± 0.28
2018	4.42 ± 1.26	0.63 ± 0.15		2.10 ± 0.45	0.74 ± 0.13	31.89 ± 38.32	16.81 ± 24.71	10.77 ± 17.08	0.80 ± 1.36	3.15 ± 4.25	0.36 ± 0.56
**T-test**											
Rainy × Dry	*p* < 0.05	*p* < 0.01		*p* < 0.01	*p* < 0.01	*p* < 0.05	*p* < 0.01	*p* > 0.05	*p* > 0.05	*p* > 0.05	*p* > 0.05
Wetland × River	*p* < 0.01	*p* < 0.01		*p* > 0.05	*p* > 0.05	*p* < 0.01	*p* < 0.01	*p* < 0.01	*p* < 0.01	*p* < 0.01	*p* < 0.01
2017 × 2018	*p* > 0.05	*p* > 0.05		*p* > 0.05	*p* > 0.05	*p* > 0.05	*p* > 0.05	*p* < 0.01	*p* > 0.05	*p* > 0.05	*p* > 0.05

There was significant variation between the hydrological period and habits on the Margalef index during the study period. The results of T-test showed that the hydrological period (*p* < 0.05) and habits (*p* < 0.01) differences were both significant (Table 2). The Margalef index of the interconnected aquatic habits was 4.01 ± 0.99 in dry season and 4.84 ± 1.37 in rainy season, respectively (Fig. 3). The mean value of Margalef index of the wetland samples (4.97 ± 1.13) was significantly higher (*p* < 0.01) than that of the river samples (3.67 ± 0.87). The result revealed that the increased hydrological connectivity promoted the richness in phytoplankton community. The spatial distribution of phytoplankton Pielou index (evenness) was very similar with that of Margalef index (richness). There was a significant interaction between the effects of hydrological period and habits on the Pielou index (Table 2, Fig. 4) (*p* < 0.01). The Pielou index of the interconnected aquatic habits was 0.56 ± 0.14 in dry season and 0.71 ± 0.17 in rainy season, respectively (Fig. 3). Unlike Margalef index, the high mean value of Pielou index were found in river samples (0.78 ± 0.12), which had a vital variation compared with that in wetland samples (0.73 ± 0.13) (*p* < 0.01). The Pielou index is a signal to indicate community species evenness^21^. Our result showed that increased hydrological connectivity is beneficial for species evenness in interconnected habits. Consist with Margalef index and Pielou index, there was significant variation on Shannon–weaver index (species diversity) in differ hydrological periods (Table 2). However, T-test showed that the habits had a weakly influenced on Shannon–weaver index (Table 2) (*p* > 0.05). The Shannon–weaver index of the interconnected aquatic habits was as low as 1.74 ± 0.40 in dry season and as high as 2.29 ± 0.74 in rainy season (Fig. 5). The spatial distribution of the Simpson index (Fig. 6) in both hydrological period and habits were very similar to that of the Shannon–Weaver index (Fig. 6). There was significant variation on Simpson index in different hydrological period (*p* < 0.01), meanwhile, the habits differences in the Simpson index were not that significant (*p* > 0.05). The correlation analysis showed that the Shannon–weaver index in all samples and rainy season samples were significantly positively correlated with the Margalef, Simpson and Pielou indices (*p* < 0.05, or *p* < 0.01) with the correlation coefficients of 0.399, 0.785, and 0.773 respectively (Table 3). In summary, Margalef index showed a weak correlation with the other three indices in all hydrological period (*p* > 0.05), but had a significant negative correlation with the Pielou index in rainy season (*p* < 0.01). Pielou index and Simpson index had a similar characteristic, which mostly significant positive correlated with Shannon–weaver index, and weakly positive correlated with Margalef index.

**Table 3:** The correlation coefficients found by correlation analysis of phytoplankton diversity indices and TSI in All samples, Wetland samples (S01–S10), River samples (S11–S17) in different hydro periods.

Sample sites	Margalef			Pielou			Simpson		
	All	Rainy	Dry	All	Rainy	Dry	All	Rainy	Dry
**Shannon–weaver**									
Correlation	0.399**	0.459**	0.215	0.785**	0.427*	0.917**	0.773**	0.873**	0.654**
Sig	0.001	0.012	0.223	0	0.012	0	0	0	0
**Margalef**									
Correlation				-0.171	-0.497**	-0.151	0.16	0.064	-0.02
Sig				0.163	0.003	0.395	0.19	0.721	0.395
**Pielou**									
Correlation							0.712**	0.758**	0.623**
Sig							0	0	0

*Denotes *p* < 0 .05 (two-tailed), **Denotes *p* < 0 .01 (two-tailed).

### The correlation between phytoplankton diversity indices and trophic states

Correlation analysis showed that the TSI in all samples was significantly negatively correlated with the Shannon–weaver index (r = -0.498), Pielou index (r = -0.545), and Simpson index (r = -0.357), but weakly positively correlated with the Margalef index (r = 0.128) (Table 4). Additionally, correlation analysis in different samples based on different hydrological period and habits was conducted. The result of correlation analysis between phytoplankton diversity indices and TSI in rainy season samples was similar with all samples's (Table 4), which significant negatively correlate with Shannon–weaver index(r = -0.498), Pielou index (r = -0.655) and Simpson index (-0.436), but weakly positively correlated with the Margalef index(r = 0.304). Conversely, all of four diversity indices had no significant correlation with TSI in dry season samples (Table 4). In wetland samples, correlation analysis showed a negatively correlation between TSI with all of four diversity indices, although had no significant coefficient. In wetland samples, TSI had a significant negatively correlated with Pielou index(r = -0.457) and Simpson index (r = -0.666).

**Table 4:** The correlation coefficients found by correlation analysis of phytoplankton diversity indices and TSI in All samples, Wetland samples (S01–S10), River samples (S11–S17) in different hydro periods.

	Margalef	Shannon–weaver	Pielou	Simpson
**All**				
Correlation with TSI	0.128	−0.498**	−0.545**	−0.357**
Sig	0.299	0.000	0.000	0.003
**Rainy season**				
Correlation with TSI	0.304	−0.498**	−0.655**	−0.436**
Sig	0.080	0.000	0.000	0.010
**Dry season**				
Correlation with TSI	0.239	−0.154	−0.289	−0.384
Sig	0.173	0.385	0.098	0.025
**Wetland**				
Correlation with TSI	−0.250	−0.307	−0.275	−0.285
Sig	0.119	0.054	0.086	0.075
**River**				
Correlation with TSI	−0.704	−0.289	−0.457*	−0.666**
Sig	0.015	0.136	0.015	0.000
**2017**				
Correlation with TSI	0.291	−0.348*	−0.607**	−0.573**
Sig	0.095	0.043	0.000	0.000
**2018**				
Correlation with TSI	−0.076	−0.417*	−0.501**	−0.473**
Sig	0.670	0.014	0.003	0.005

*Denotes *p* < 0.05 (two-tailed), **Denotes *p* < 0.01 (two-tailed).

## Discussion

Phytoplankton diversity indices had been shown to be a useful approach in the evaluation of community evenness, richness, function and stability^3^. Particularly, phytoplankton alpha diversity indices had also been applied as a tool for trophic state assessment and regulate the potential harmful algae blooms^12,21^. The main advantage of phytoplankton alpha diversity indices assessment ecological trait is which included the ecological information of richness, abundance and evenness^3,8^. In this study, we hypothesized that the phytoplankton alpha diversity indices has the potential to provide a signal linking trophic state variation in hydrologically connected aquatic habitats. Our results showed that the phytoplankton alpha diversity indices and environmental parameters presented an obvious variation in differ hydrological periods. In addition, the phytoplankton alpha diversity indices had a closely relation with trophic states index. All of these findings verified our hypothesis that the phytoplankton alpha diversity indices were a potential signal for indicating trophic state variations in hydrologically connected aquatic habitats.

### Impacts of hydrological connectivity on phytoplankton communities

The Songhua River Basin was one of the earliest urbanized centers in China since the 1950s, which with many functions such as household, industrial, and agricultural water use^23^. Former studies had showed that the Songhua River had been polluted in different degrees except the river's source^15^. However, the study focus on environmental filters on plankton community assembly is little, particular in hydrologically connected aquatic habits^16^. In present study, we developed the phytoplankton community matrices in hydrologically connected aquatic habits of the Harbin Section of the Songhua River firstly. Hydrological connectivity is changed by regulation of water flow, which results in fluctuations in environmental parameters, such as DO, EC, and nutrient concentrations^2^. In present study, the nutrient parameters and TSI showed a significant temporal difference (*p* < 0.05 or *p* < 0.01) in two habits, which were generally higher in dry season (low hydrological connectivity) and lower in the rainy season (high hydrological connectivity) (Table 1). TP and TN resuspension are closely related to hydropexis in lentic aquatic system^24^. Thus, the increased nutrient concentrations in dry season were most likely due to lower hydrological connectivity. In study period, we found the phytoplankton community was mainly contributed by Cyanophyta and Bacillariophyta. The phytoplankton dynamics in study area are similar to those of small European and African interconnected habits such as Lake Naivasha^25^, Lake Mikri Prespa^12^, and Mississippi River^26^. Environmental variables such as flow rate, velocity, light and nutrient availability are vital characteristics that directly affect the diversity and dispersal of phytoplankton in rivers and wetlands^2,21,27^. Thus, the dynamic of phytoplankton community can be an excellent bio-indicator in spatial and temporal scale of this environmental change^2,28^. The hydrological connectivity in wetland-river conditions was significantly affected by rainfall and snowmelt events^29^. In general, rainfall promotes hydrological connectivity in freshwater ecosystem^30^. Raffoul et al.^25^ noted that hydrological connectivity can strongly influence phytoplankton community and ecological traits through the interchange of nutrients and organisms in interconnected aquatic habitats. In present study, phytoplankton abundance showed significantly differences (*p* < 0.05, or *p* < 0.01) in two hydrological period of the interconnected habits (Table 2), with higher values mostly recorded in dry season and lower values in rainy season. In spatial, the total phytoplankton abundance of wetland was strongly higher than that of river, coinciding with increaseing in the concentrations of TN. This indicates the most dynamic characteristics of phytoplankton community of the interconnected aquatic habits are closely linked to trophic state fluctuation, which agrees with observations by Hu et al.^31^ and Dijkstra et al.^32^. In general, diatoms are considered as a vital component of phytoplankton communities in wetlands and rivers being typically the dominant taxonomic group in terms of species richness in such environments^33^. Compared with Chlorophyta and Euglenophyta species, diatoms have a great ability to tolerate extreme conditions such as low water temperature^34^. Reynolds recorded that the parts of benthic Diatoms (*Aulacoseira* spp., *Cyclotella* spp.) were sensitive to flushing and riptide^35^. The high hydrological connectivity in rainy season increased the flushing intensity and disturbance the stability of aquatic conditions^30^. Compared with the relative lower diatoms abundance in rainy season (6.2 ± 5.5 × 10^6^ ind./L) (*p* < 0.01), we found a significant increase in dry season (19.53 ± 25.34 × 10^6^ ind./L), which may be due to the variation of water flushing in high hydrological connectivity, which consist with Waite et al.^36^. Be contrary to diatoms, unexpected, there was no significant variation of abundance of Cyanophyta in the interconnected habits during differ hydrological period. Katsiapi et al. (2020) noted that the motile species of Cyanophyta and Euglenophyta were dominated in connectivity lakes^12^. Cyanophyta (*Pseudanabaena* spp.) could tolerate mixing regime and sensitive to low nitrogen^37^. In this study, we found mean TN ranged from 0.40 ± 0.33 mg/L to 1.55 ± 0.83 mg/L, indicating severe eutrophication. In addition, our results showed that TN had a significant spatial differences (*p* < 0.01), which were generally higher in lower hydrological connectivity period (Table 1). The weakly influencing of hydrological connectivity on Cayanophyta species was mainly attributed to individual nutrient available and adversity adaptive strategy^37^. These results consisted with Yuan et al.^2^, which environmental variables affecting phytoplankton community differed under different hydrologic regimes. In summary, the lentic environment is very important for the production, diversity, relative abundance, and fecundity of parts phytoplankton in interconnected habits. In general, phytoplankton alpha diversity pattern was linked to hydrological connection, wind, and grazing in freshwater ecosystem^38^. In further study, the study focus on multivariate factors included “bottom-up” and “top-down” were necessary.

### Phytoplankton diversity indices response to trophic state variation in interconnected habitats

Phytoplankton communities in the rivers are usually assumed to be mixed on local scales unless intensity disturbance lead to discontinuities separating water^39^. Recent studies suggested that hydrological features variations leading subtle discontinuities may be vital for influencing phytoplankton alpha-diversity patterns^8,10,21,40^. In this study, based on spatial interpolation analysis approach and correlation analysis we found that hydrological connectivity was a considerable factor to dynamic phytoplankton alpha diversity pattern in small spatial scale. Our results showed that alpha diversity was significantly higher in rainy season compared to that in dry season, suggested that high hydrological connectivity can promote the richness and evenness in phytoplankton community, which agree with Yuan et al. (2018). Hydrological connectivity is improved with an increase in rainfall, as well as promoted the water exchange in connected habits^30^. Water exchange carries biotic and abiotic materials, including nutrients, and phytoplankton into interconnected habitats, accelerated an increase in phytoplankton diversity and evenness within interconnected habitats^24,38,41^. In both marine and freshwater ecosystems, environmental conditions play a vital role in determining local taxonomic diversity. Alpha diversity is considered positively dependent on environmental filter in local scale^41^. The increased of Shannon–weaver, Simpson and Pielou index were mainly be consider as a signal of community stability and improved trophic status^21,42^. In this study, we found Shannon–weaver, Simpson and Pielou index were negatively significant correlate with TSI, particular in rainy season presented a high correlation coefficient (Table 4) (*p* < 0.01). Notable, there was no significant correlation between Shannon–weaver, Simpson and Pielou index in dry season. This result indicating that the disturbance of hydrological connectivity could promote organism and nutrient transport of internal hydrodynamics in different habitats, which probably be an important reason for dynamic on the phytoplankton community assembly^10,43^. Contrary to our expected, there was weakly positive correlation between Margalef index and TSI in two connected aquatic habits. Although there was a clear decreaseof Margalef index were found (*p* < 0.05), with significant increase of TSI (*p* < 0.01), however, correlation analysis showed weakly positive correlation between Margalef index and TSI (*p* > 0.05) in differ hydrological periods. In common, low connectivity with high trophic stats was provided an appropriate condition for colonization of pioneer species^33,44^. Phytoplankton communities were mainly characteristic by simplex richness and predominated by contender species in eutrophic lakes and rivers ecosystem^25,45^. Margalef index is recorded as a signal for indicating the richness of community in aquatic and terrestrial ecosystem^21^. In fact, Margalef index was advantage in focusing on richness and taxonomic composition, as well ignoring the abundance information of community. Even so, we found that the hydrological connectivity in the two aquatic habitats appeared to weakly influence on phytoplankton richness through the exchange of water and nutrients, which agrees with observations by Yuan et al. (2018). The environmental conditions in Jinhewan Wetland such as shallow water and high trophic state created a unique habitat and provided good conditions for the reproduction of phytoplankton. Our study revealed that Margalef and Pielou indices presented a significant different in interconnected habits of this study (Table 4). This finding demonstrated that the environmental filter of interconnected aquatic habits was relative weakly on alpha diversity indices in the same hydrological period. Prior studies showed that the phytoplankton diversity indices wa not a relevant tool to ecological assessment, due to difficult distinguish between different levels of trophic states (such as oligotrophic, meso-trophic and eutrophic). Yang et al. (2016) noted that univariate phytoplankton diversity index descriptive trophic state in eutrophic lake is unreliable^21^. The efficiency and stability of trophic state assessment schemesis were vital related to nutrient condition, biotic matrices, and survey scale^41,46,47^. Thus, an integrated index of nutrients and phytoplankton communities to assess rivers trophic states is necessary. Compared to single factors such as chemical factors, biomass or appear of indicator species, diversity indices are more integrated and relevant assessment approach with vital complete ecological information. Phytoplankton diversity indices were deemed to effective reflect the response of biotic matrices to environmental change in lakes or reservoirs ecosystem^21,48^. However, the application of phytoplankton diversity indices in trophic states assess of rivers is still weak. To further utilize phytoplankton diversity indices to assess ecological health in rivers, the hydrological factors (flow velocity), larger scale, and precise sample frequency were necessary. In present study, the phytoplankton indices assessment schemes were performed combine with alpha diversity indices and TSI. In addition, we inferred that the relevant phytoplankton indices assessment schemes could be got by a constrained ordination and GIS-based approach for visualizing the interaction of phytoplankton diversity and nutrient concentrations through multivariate statistical methods. Our study provides evidence that hydrological connectivity contribute an important part of the theory of environmental filter. Understanding the relationships between diversity indices and trophic state aspects remains a challenge in hydrologic research, and at the same time, it is essential for establishing water management database in larger spatial and temporal scale.

## Conclusions

The feasibility of phytoplankton alpha diversity to serve as biological indicator for assessing trophic states in interconnected habits were investigated in the present study, based on the correlation analysis between TSI and phytoplankton alpha diversity indices including Shannon–Weaver index, Margalef index, Simpson index and Pielou index. The multivariate statistical analysis revealed that hydrological connectivity strongly influenced the phytoplankton alpha diversity indices and trophic states in different hydrological period. In spatial, the environmental filter was relative weakly on phytoplankton alpha diversity indices in interconnected habits during the same hydrological period. The development of integrated phytoplankton diversity indices is benefit for environmental regulate in hydrologically connected aquatic habits.
